# Measuring core temperature using the proprietary application and thermo-smartphone adapter

**DOI:** 10.1007/s10877-016-9968-8

**Published:** 2016-12-24

**Authors:** Tomasz Darocha, Jacek Majkowski, Tomasz Sanak, Paweł Podsiadło, Sylweriusz Kosiński, Kinga Sałapa, Piotr Mazur, Mirosław Ziętkiewicz, Robert Gałązkowski, Łukasz Krzych, Rafał Drwiła

**Affiliations:** 1 Severe Accidental Hypothermia Center, Kraców, Poland; 2 Department of Anesthesiology and Intensive Care, John Paul II Hospital, Severe Accidental Hypothermia Center, 80 Pradnicka St., 31-202 Kraców, Poland; 3 Heart for Life Foundation, Kraców, Poland; 4 Institute of Cardiology, Jagiellonian University Medical College, Kraców, Poland; 5 Polish Medical Air Rescue, Warsaw, Poland; 6 Department of Disaster Medicine and Emergency Care, Jagiellonian University Medical College, Kraców, Poland; 7 Department of Combat Medicine, Military Institute, Warsaw, Poland; 8 Polish Society for Mountain Medicine and Rescue, Szczyrk, Poland; 9 Department of Anesthesiology and Intensive Care, Pulmonary Hospital, Zakopane, Poland; 10 Poland Tatra Mountain Rescue Service, Zakopane, Poland; 11 Department of Bioinformatics and Telemedicine, Jagiellonian University Medical College, Kraców, Poland; 12 Department of Cardiovascular Surgery and Transplantology, John Paul II Hospital, Kraców, Poland; 13 Department of Emergency Medical Services, Medical University of Warsaw, Warsaw, Poland; 14 Department of Cardiac Anaesthesia and Intensive Care, Medical University of Silesia, Katowice, Poland

**Keywords:** Accidental hypothermia, Core temperature, Measurement, Thermometer, Equipment

## Abstract

Fast and accurate measurement of core body temperature is crucial for accidental hypothermia treatment. We have developed a novel light and small adapter to the headset jack of a mobile phone based on Android. It has been applied to measure temperature and set up automatic notifications (e.g. Global Positioning System coordinates to emergency services dispatcher, ECMO coordinator). Its validity was confirmed in comparison with Vital Signs Monitor Spacelabs Healthcare Elance 93300 as a reference method, in a series of 260 measurements in the temperature range of 10–42 °C. Measurement repeatability was verified in a battery of 600 measurements (i.e. 100 readings at three points of 10, 25, 42 °C for both esophageal and tympanic catheters). Inter-method difference of ≤0.5 °C was found for 98.5% for esophageal catheter and 100% for tympanic catheter measurements, with concordance correlation coefficient of 0.99 for both. The readings were almost completely repeatable with water bath measurements (difference of ≤0.5 °C in 10 °C: 100% for both catheters; in 25 °C: 99% for esophageal catheter and 100% tympanic catheter; in 42 °C: 100% for both catheters). This lightweight adapter attached to smartphone and standard disposable probes is a promising tool to be applied on-site for temperature measurement in patients at risk of hypothermia.

## Introduction

Accidental hypothermia is a life-threatening condition that requires specific management. Patient’s core body temperature (Tc) is a crucial piece of information, necessary for the correct treatment [1–3] The hand-held monitors allowing Tc measurement in prehospital settings are usually too large and too heavy for mountain rescue. These low-reading devices are usually expensive, and they are not a part of standard Emergency Medical Services or Mountain Rescue Services equipment. On the other hand, the peripheral thermometers do not have acceptable accuracy to estimate core body temperature [4]. Thus, neither the accurate Tc assessment, nor the correct management of hypothermic patient, are possible nowadays in the pre-hospital setting. This prompted us to develop a lightweight, low-reading thermometer based on a smartphone with Android operating system, and certified single use esophageal/epitympanic/bladder/rectal probes. This concept provides patient’s safety, low costs and wide availability of the device.

## Materials and methods

The adapter (15 cm × 2 cm × 1.5 cm, 35 g) for the headphone jack of a smartphone based on Android operating system and a dedicated application have been developed. The purpose of the adapter is to connect a mobile phone with widely available disposable catheters to measure temperature using the YSI 400 jack (e.g. Smiths Medical, Covidien, DeRoyal, Starboard Medical). It is possible to connect disposable catheters for measuring the temperature of the esophagus, rectum, tympanic membrane, and urinary bladder. Application implements proprietary signal processing algorithm, providing means of measuring resistance, and thus the temperature of the probes. It allows re-using of hardware available in every smartphone (electronics for signal processing, battery, display) for purpose of those measurements, effecting in low-cost adapter. Mathematic and physic basis for this algorithm, as well as implementation of hardware (adapter) and software parts are beyond the scope of this paper—it focuses on showing proof-of-concept compared to reference techniques. The time for a single temperature measurement for the proprietary system developed is 120 s. The discharge of the smartphone battery in a single measurement is comparable to a 2 minutes’ phone call.

In the first stage of this study, temperature measurements were performed using the smartphone with the developed system in comparison with Vital Signs Monitor Spacelabs Healthcare Elance 93,300 with the range of 5.0–50 °C and the accuracy of ±0.1 °C plus the accuracy of the catheter to measure the temperature. The following catheters were used in the experiment: Smiths Medical Level 1 Esophageal/Rectal 12 Fr Temperature Probe 400 series with the accuracy of ±0.2 °C and Smith Medical Level 1 Tympanic Temperature Sensor 400 Series with the accuracy of ±0.2 °C. The catheters were simultaneously submerged in a water bath at equal distances. The LW 502 AJL Electronic water bath was used as a reference with temperature adjustment between 10 and 62 °C and the accuracy of temperature stabilization up to 0.2 °C. In this step, two temperature measurements in the range of 10–42 °C every 0.5 °C were carried out for the esophageal and tympanic catheter connected to the testing system and the above-mentioned Spacelabs’. A total of 260 measurements were carried out (130 temperature measurements using the testing system and 130 temperature measurement using Spacelabs’).

In the next step, temperature measurements based on the test system were carried out using the water bath at three points of 10, 25, 42 °C (100 readings at each temperature) for each kind of esophageal and tympanic catheter. Hence, the total of 600 measurements were taken in this step, and 860 temperature measurements throughout the experiment.

## Statistical methods

Agreement between two continuous data measured from two different measurement methods was evaluated by means of concordance correlation coefficient (CCC) [5]. It measures the variation of their linear relationship from the 45° line through the origin. Pearson correlation coefficient (PCC) may be very misleading in such situations. Any departures from this line would produce CCC below one even if PCC is equal to one [5, 6]. CCC was estimated by variance components to reduced bias of the moment-methods estimator proposed by Lin [7]. Furthermore, the analysis of limits of agreement was applied proposed by Bland and Altman [8, 9]. The idea of this analysis is to plot the individual difference between measurements taken from two different methods against their individual mean to investigate the size of discrepancies between two measurements and judge whether they are clinically acceptable or not. Clinically acceptable limits of agreement were *a priori* defined to be ±0.5 °C. It is assumed that there is no relation between the differences and the mean and that the differences are normally distributed [9]. In case of lack of independence a logarithmic transformation was applied to the raw data. Because this transformation was unsuccessful the alternative analysis proposed by Bland and Altman was applied [8–10], i.e. least squares regression to predict the measurement obtained by the old methods from the measurement obtained by the new one, and calculate 95% prediction interval for the old methods depending on the various temperatures measured by the new one. Bonferroni correction was applied to calculate prediction limits for few new observations. This approach gives results similar to the limits of agreement [10]. The assessment of relationship between the individual differences and means was based on Spearman rank correlation coefficient because both features were not normally distributed. The Shapiro–Wilk test was applied to check normality. One-sample Wilcox test was performed to verify whether the median temperature was equal to certain water bath temperature.

Results with p-value less than α = 0.05 were considered as statistically significant. R software was used to statistical analyses.

## Results

**Fig. 1:**
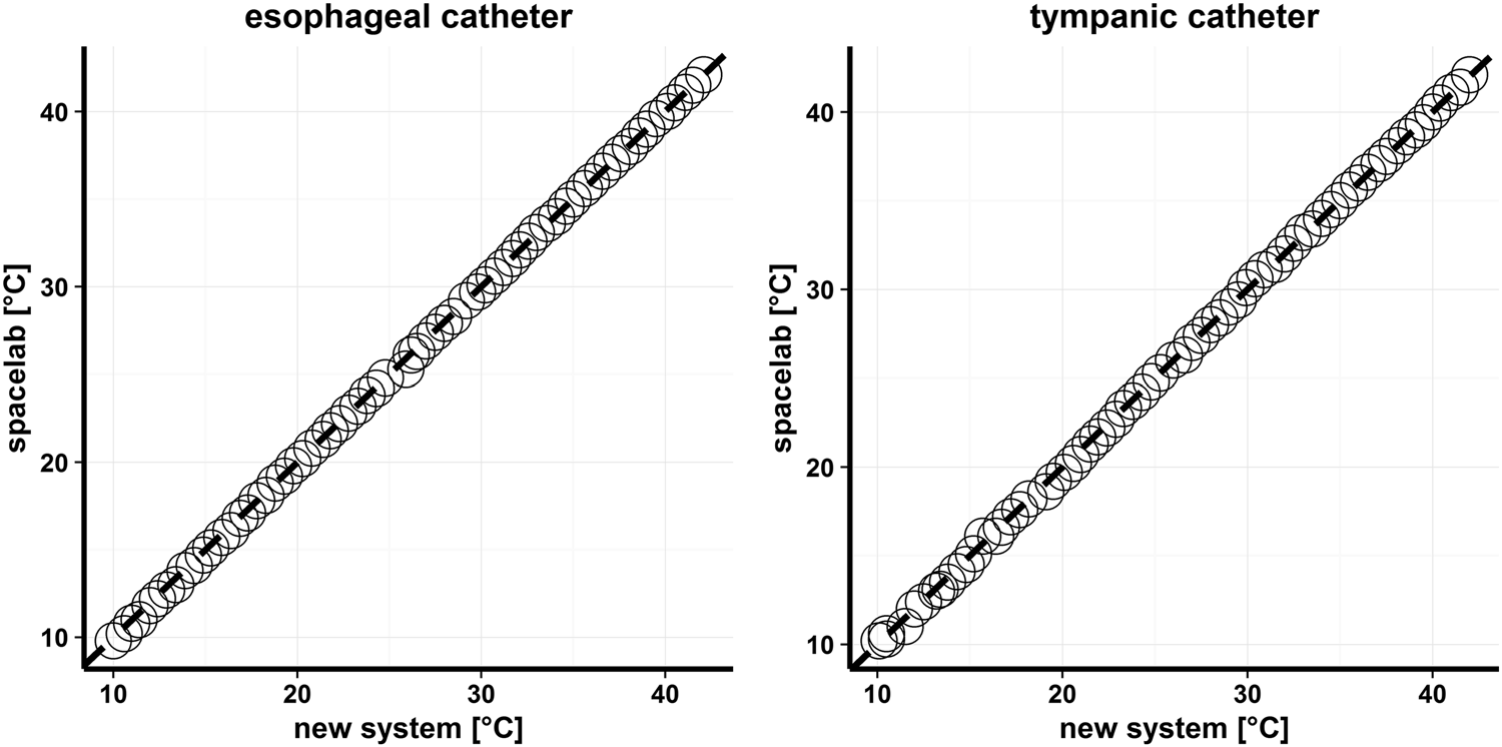
New system measurements versus spacelab measurements for esophageal catheter (*left panel*) and for tympanic catheter (*right panel*). *Dashed lines* represent the 45° line through the origin

**Fig. 2:**
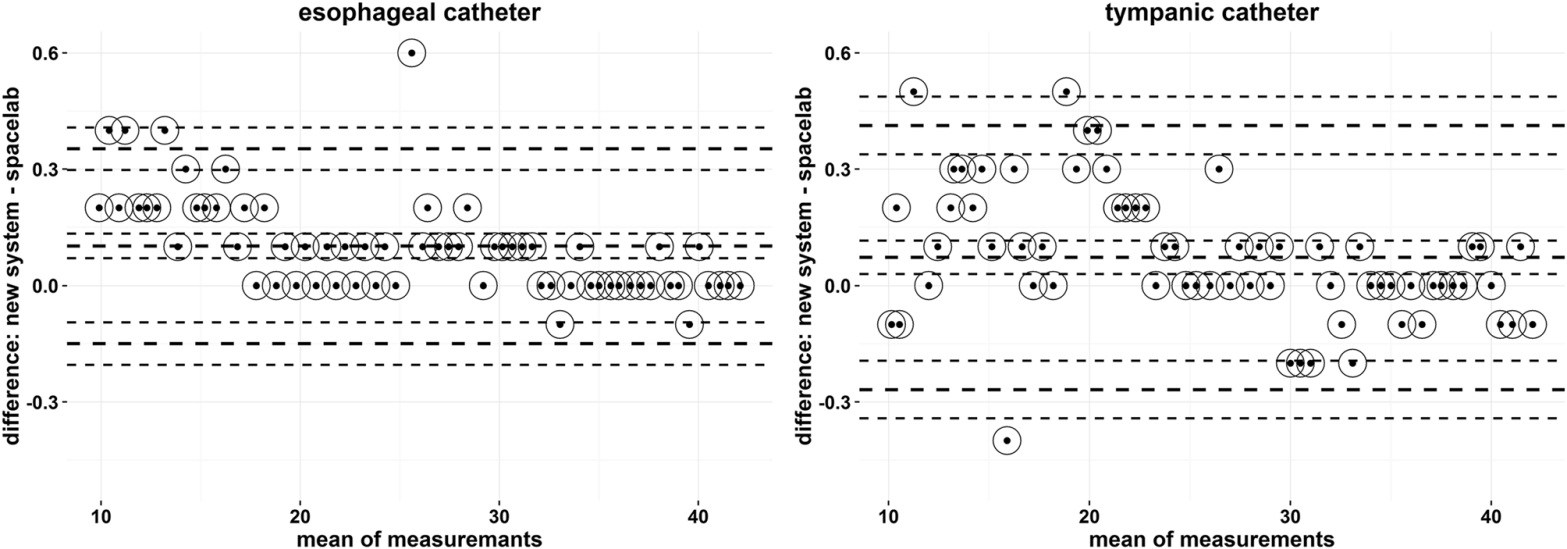
Bland–Altman plots for esophageal catheter (*left panel*) and for tympanic catheter (*right* panel)

Firstly, agreement between readings from new system and the Spacelabs were carried out, separately for the esophageal catheter (EC) and the tympanic catheter (TC). The differences between new system and spacelab measurements were less or equal to 0.5 °C in 64 of 65 cases (98.5%) for EC, and in all cases (100%) for TC (Table 1). New system measurements are plotted against the spacelab measurements in Fig. 1 (EC: left panel, TC: right panel). It can be seen, within a tolerable error, that the measurements fall on the 45° line through the origin in both cases. This indicates that the spacelab is highly reproducible by the new system. The concordance coefficient with 95% confidence interval is equal to 0.9999 (0.9998, 0.9999) and 0.9998 (0.9997, 0.9999), respectively for the esophageal and tympanic catheter. The left panel of Fig. 2 presents comparison of measurements by means of the limits of agreement plot for esophageal catheter. The mean difference was 0.102 (95% CI 0.069, 0.133), hence, the new system overestimated the measurement of temperature, by between 0.069 and 0.133. The limits of agreement lied in the range of −0.149 (95% CI −0.204, −0.095) and 0.353 (95% CI 0.298, 0.408). The right panel of Fig. 2 presents the limits of agreement plot for the tympanic catheter. The mean difference was 0.073 (95% CI 0.029, 0.115), hence, the new system tended to give a higher measurement of temperature, but only by between 0.029 and 0.115. The limits of agreement lied in the range of −0.268 (95% CI −0.343, −0.195) and 0.413 (95% CI 0.338, 0.487). These graphs should be interpreted with caution because of the differences are not normally distributed (p < 0.001 in both cases) and the differences are not independent on the mean, i.e. the individual differences decrease as the averages of measurements increase (EC: R = −0.64, p < 0.001, TC: R = −0.45, p < 0.001). The logarithmic transformation was applied to the raw data but without expected effects. Owing to this, the regression analysis was applied to describe the negative trend and to calculate 95% prediction intervals for the spacelab given the various temperatures measured by the new system for both the esophageal and the tympanic catheter. It was found that the measurements of the spacelab for the esophageal catheter lied between 9.50 and 10.04 °C with probability of 0.95 when the new system reading was equal to 10 °C, between 19.58 and 20.12 °C when the new system was 20 °C, between 29.66 and 30.20 °C when the new system was 30 °C and between 39.74 and 40.28 °C when the new system was 40 °C. Thus, the presented prediction intervals for the spacelab were within the limit of ±0.54 °C. Next, the measurement of the old method for the tympanic catheter lied between 9.38 and 10.22 °C with probability of 0.95 when the new method reading was equal to 10 °C, between 19.47 and 20.29 °C when the new system was 20 °C, between 29.55 and 30.37 °C when the new system was 30 °C and between 39.62 and 40.45 °C when the new system was 40 °C. Thus, the presented prediction intervals for spacelab were within the limit of ±0.82 °C.

**Table 1:** Results of comparison the new system and spacelab measurements

	Esophageal catheter	Tympanic catheter
New system = spacelab ± 0.5 °C	64 (98.5%)	65 (100%)
New system > spacelab	38 (58.5%)	32 (49.2%)
New system = spacelab	25 (38.5%)	20 (30.8%)
New system < spacelab	2 (3.1%)	13 (20%)

Results presented as numbers and percentages

The next step of this study was to compare temperature measurements taken from the new system and the reference temperature of water bath at three points of 10, 25, 42 °C (100 readings of the new system at each temperature) for each kind of esophageal and tympanic catheter. Descriptive statistics of measurements are presented in Table 2. The mean of new system readings did not significantly differ from the reference 10 °C water bath (EC: p = 0.215, TC: p = 0.773), but significantly differ from 25 °C (EC, TC: p < 0.001) and from 42 °C (EC, TC: p < 0.001).

**Table 2:** Descriptive statistics of new system in comparison with water bath

Water bath temperature	Esophageal catheter	Tympanic catheter
10 °C		
Mean ± SD	10.01 ± 0.10 °C	10.01 ± 0.17 °C
Me (Q_1_ – Q_3_)	10 (10–10)°C	10 (10–10)°C
Min–max	9.8–10.3 °C	9.9–10.1 °C
New system = water bath ± 0.5 °C	100 (100%)	100 (100%)
New system > water bath	21 (21%)	2 (2%)
New system = water bath	56 (56%)	97 (97%)
New system < water bath	23 (23%)	1 (1%)
25 °C		
Mean ± SD	24.69 ± 0.08 °C	24.59 ± 0.13 °C
Me (Q1 – Q3)	24.7 (24.6–24.7)°C	24.6 (24.5–24.6)°C
Min–max	24.3–25 °C	24.5–25.1 °C
New system = water bath ± 0.5 °C	99 (99%)	100 (100%)
New system > water bath	0 (0%)	3 (3%)
New system = water bath	2 (2%)	1 (1%)
New system < water bath	98 (98%)	96 (96%)
42 °C		
Mean ± SD	42.11 ± 0.13 °C	42.13 ± 0.85 °C
Me (Q1 – Q3)	42.1 (42.1–42.2)°C	42.1 (42.1–42.2)°C
Min–max	41.9–42.5 °C	41.9–42.4 °C
New system = water bath ± 0.5 °C	100 (100%)	100 (100%)
New system > water bath	75 (75%)	81 (81%)
New system = water bath	14 (14%)	18 (18%)
New system < water bath	11 (11%)	1 (1%)

Continuous measurements are presented as mean with standard deviation (SD), median (Me) with lower (Q1) and upper (Q3) quartile, and minimum and maximum value. Categorical data (i.e. results of comparison new system with water bath) are presented as numbers and percentages

## Discussion

Our smartphone-based thermometer provides the possibility of accurate core body temperature measurement at initial management, that can be life-saving for hypothermic patients. This study confirms the satisfying accuracy of temperature measurement, sufficient for therapeutic decisions. Inaccuracies which were found (avg. 0.1 °C) are not clinically important.

The treatment of a patient in accidental hypothermia should be modified according to patient`s core body temperature. This refers to the acceptability of a physical activity in mild hypothermia, careful passive transport in severe hypothermia and other therapeutical decisions. European Resuscitation Council (ERC) indicates the necessity of the defibrillations number and the drugs dosage reduction when Tc is below 30 °C [2]. Low body temperature (below 30 °C) is also an indication for continuing cardiopulmonary resuscitation (CPR) in asystolic snow avalanche victims [2]. The early hypothermia recognition allows to perform suitable management and to reduce mortality among trauma patients. Appropriate insulation and warming can limit cold-related coagulopathy and blood loss [11–13]. ERC and Wilderness Medical Society guidelines allow to intermit CPR (if necessary) in severely hypothermic victims during evacuation.

According to ERC guidelines, patients with Tc below 28 °C in cardiac arrest or cardiac instability, should be transported to Extracorporeal Life support (ECLS) centre (ECMO is preferred) [2]. Accurate Tc measurement is required to fulfil guidelines mentioned above.

The “gold standard” for Tc assessment is esophageal temperature measurement. In alert hypothermic patients, epitympanic temperature (thermistor, non-IR) is also accepted [2, 14]. However, not many rescue services are equipped with appropriate thermometers.

Karlsen et al. have described the equipment of different rescue services in Norway, especially the devices designed to prevent, recognize and treat accidental hypothermia, and showed that as little as 12% of ground ambulances have the opportunity to read the core temperature [15]. In Poland, Tc measurement is not possible in ground ambulances, but 28% of air rescue teams have appropriate thermometers [Darocha, unpublished data].

It is necessary to equip the ambulance/helicopter rescue teams, mountain rescue teams and search and rescue service with thermometers designed to measure core temperature. Most of such devices, however, are expensive and enlarge the weight of equipment (which is not desirable in mountain rescue settings). The Swiss Staging System is an alternative solution for rescue teams and allows to estimate Tc solely upon clinical symptoms. Unfortunately, this very useful and simple method can lead to dangerous mistakes, when hypothermia is associated with trauma or alcohol abuse. Differentiated susceptibility to cooling process can also interfere with the clinical temperature assessment. As a consequence, it can lead to (faulty) withholding of defibrillation or to excessive ventilation resulting in ventricular fibrillation precipitation [16]. Pasquier et al. have presented the homemade thermometer adapted to the esophageal temperature measurement in the field settings. Unfortunately, their device consists of an uncertified probe (without the homologation for medical use) [17].

Nowadays, smartphones are very common and the rescue teams are usually equipped with them. A thermometer constructed as smartphone-based device is easily available in prehospital settings. In case of malfunction or battery exhaustion, it can be quickly replaced by another one. Moreover, the use of medically certified probes assures the patient’s safety. The probes are relatively cheap and easy to replace, thus the triage of snow avalanche victims can be quickly performed.

There are additional functions of the application that can help a rescue team The software allows to send automatically preset data, such as GPS coordinates to a rescue dispatch center or patient’s Tc to preferred hospital/ECMO coordinator. These features can reduce the burden of rescuers and on-scene time [18]. The application can also display the algorythm of patients management, adjusted to the measured temperature. The on-screen prompts can be useful support for rescuers in a stressful situation. This concept is based on the idea of checklists successfully used in aviation emergency scenarios [19].

The specificity of rescue operations in various settings necessitates the maximizing of the results that can be achieved with the least amount of hardware. Today, every smartphone in the pocket of a rescue worker is an unused computer which can serve for diagnosis, transmitting or teleconsultation.

The main limitation of our study is the lack of accuracy control in the field settings, especially in cold environment. However, using the same probes as professional thermometers, we do not expect significant differences. The accuracy and reliability of the whole system (probe, connector and phone) in cold and humid conditions require further studies. Another point to improve is the measurement time. It probably depends on Android version and phone hardware.

In conclusion, we have developed and presented a lightweight adapter to measure deep temperature using a smartphone and certified disposable probes for hypothermia patients. This design ensures patient safety, low cost and ready availability of equipment. The capability to set up automatic notifications (temperature value to the chosen hospital or ECMO coordinator as well as GPS coordinates to the emergency services dispatcher) reduces the number of steps to be taken by the rescue workers and in this way it can potentially shorten on-scene time. Patient management guidelines displayed by the application pertaining to deep temperature measured are a substantial support for the rescue operations carried out under stress conditions.

## Abbreviations

Tc: Core body temperature
CCC: Concordance correlation coefficient
PCC: Pearson correlation coefficient
EC: Esophageal catheter
TC: Tympanic catheter
CPR: Cardiopulmonary resuscitation
ERC: European resuscitation council
GPS: Global positioning system
ECMO: Extracorporeal membrane oxygenation
ECLS: Extracorporeal life support
